# Impact of Vaccines Across the Lifespan: A New Perspective in Public Health—Conclusions of an Expert Panel—Part 1 †

**DOI:** 10.3390/vaccines14020183

**Published:** 2026-02-15

**Authors:** Roberto Debbag, María L. Ávila-Agüero, José Brea, Carlos Espinal, Rodrigo Romero-Feregrino, Jaime R. Torres, Hebe Vázquez, Robinson Cuadros, Gustavo Lazo-Páez, Andrea Schilling, Pablo Bonvehí, Maisa Kairalla, Alfonso J. Rodríguez-Morales

**Affiliations:** 1 Latin-American Vaccinology Society, Buenos Aires C1425AWK, Argentina; rdebbag@hotmail.com (R.D.); drbrea@hotmail.com (J.B.); 2 Pediatric Infectious Diseases Department, Hospital Nacional de Niños, San José 111221, Costa Rica; avilaaguero@gmail.com; 3 Center for Infectious Disease Modeling and Analysis, Yale University School of Public Health, New Haven, CT 06510, USA; 4 Facultad de Ciencias de la Salud, Instituto Tecnológico de Santo Domingo, Santo Domingo 10602, Dominican Republic; 5 Department of Global Health, Robert Stempel College of Public Health and Social Work, Florida International University, Miami, FL 33199, USA; caespina@fiu.edu; 6 Asociación Mexicana de Vacunología, Instituto Para el Desarrollo Integral de la Salud (IDISA), Instituto Mexicano del Seguro Social (IMSS), CONCAMIN, Saint Luke School of Medicine, Academia Mexicana de Pediatria, Av. Cuauhtémoc 271, Interior 101, Colonia Roma, Cuauhtémoc, Mexico City ZC 06700, Mexico; drrodrigo@idisalud.com; 7 Infectious Diseases Section, Tropical Medicine Institute, Universidad Central de Venezuela, Caracas 1050, Venezuela; jaimerafael.torres@gmail.com; 8 Grupo de Vacunas de la Fundación Centro de Estudios Infectológicos (FUNCEI), Buenos Aires C1425AWK, Argentina; hebevazquez@gmail.com; 9 Asociación Internacional de Gerontología y Geriatría, Comité Latinoamericano y del Caribe, Carrera 7C Bis 139-17, Bogotá 110121, Colombia; robinsoncuadros@gmail.com; 10 Universidad de Ciencias Médicas, San José 2060, Costa Rica; gustavo.lazo@gmail.com; 11 Servicio de Inmunología y Reumatología Pediátrica, Hospital Clínica Bíblica, San José 10104, Costa Rica; 12 Institute of Science and Innovation in Medicine (ICIM), Facultad de Medicina, Clínica Alemana, Universidad del Desarrollo, Santiago 7610658, Chile; dra.andrea.schilling@gmail.com; 13 Centro de Educación Médica e Investigaciones Clínicas “Norberto Quirno” (CEMIC), Buenos Aires 1430, Argentina; pablobonvehi@gmail.com; 14 Universidade Federal de São Paulo, São Paulo 04021-001, Brazil; maisakairalla@uol.com.br; 15 Faculty of Health Sciences, Universidad Científica del Sur, Lima 15067, Peru; 16 Grupo de Investigación Biomedicina, Faculty of Medicine, Fundación Universitaria Autónoma de las Américas-Institución Universitaria Visión de las Américas, Pereira 660003, Colombia

**Keywords:** immunosenescence, inflammaging, vaccine-mediated immunomodulation, immune fitness, lifelong vaccination, healthy aging, public health

## Abstract

Population aging is the most significant demographic transformation of the 21st century, reshaping health systems, economies, and societies. The biological processes of immunosenescence and inflammaging weaken host defenses, reduce vaccine effectiveness, and increase vulnerability to infectious and chronic diseases. These changes underscore the urgent need for preventive strategies that extend beyond childhood immunization. Vaccination is a cornerstone of healthy aging, capable of preventing infections and has been associated with reductions in systemic inflammation, frailty, and loss of functional independence in later life. Furthermore, new insights into vaccine-mediated immunomodulation, including trained immunity, adjuvanted formulations, and epigenetic reprogramming, highlight the evolving role of vaccines as modulators of immune fitness across the lifespan. This first part of our review examines the intersection of aging and immunity, as well as the potential of vaccines to address these challenges. Part 2 will expand on specific vaccines, proposed vaccination schedules, and global perspectives for lifelong immunization.

## 1. Introduction

**Figure 1:**
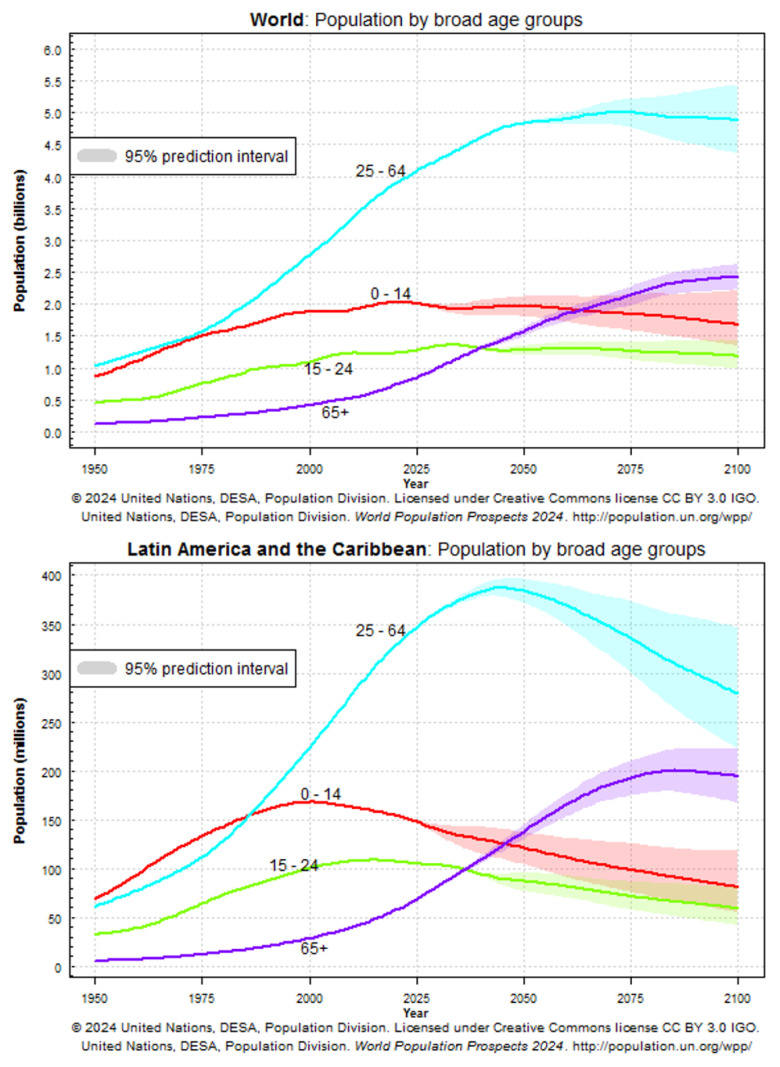
Global and regional population aging trends and their implications for public health. Projected changes in population structure by age group worldwide and in Latin America and the Caribbean, based on United Nations World Population Prospects 2024. The figure illustrates the rapid expansion of older age groups (≥60 and ≥80 years), highlighting the increasing burden of age-related chronic and infectious diseases and the growing demand for preventive strategies, including lifelong vaccination policies. Source: United Nations Department of Economic and Social Affairs, Population Division. https://population.un.org/wpp/graphs?loc=904&type=Demographic%20Profiles&category=Line%20Charts, accessed on 1 December 2025.

The 21st century has been marked by profound demographic changes, among which population aging stands as one of the most transformative and far-reaching phenomena in modern history. Unlike previous centuries, where infectious diseases and high fertility largely shaped population structures, today’s societies are experiencing a dual demographic revolution characterized by declining fertility rates and sustained increases in life expectancy [1,2,3]. These two forces have reshaped the demographic pyramid into a more rectangular profile, with a rapidly expanding share of older adults across societies worldwide. According to the World Health Organization (WHO), by 2050, more than 2.1 billion people will be aged 60 years or older, representing nearly 22% of the global population. In addition, the population aged 80 years and older is projected to triple over the same period, reaching more than 426 million (https://www.who.int/news-room/fact-sheets/detail/ageing-and-health) (accessed on 1 December 2025) (Figure 1) [4]. These figures illustrate the speed and magnitude of this demographic transformation, which is unfolding at a pace far greater than the adjustments that health systems, economies, and social structures are currently prepared to accommodate [5,6,7].

This unprecedented demographic transition, while undeniably a testament to human development, scientific progress, and public health achievements, also poses significant and complex challenges [8,9]. On the one hand, it reflects remarkable advances in medical technology, infectious disease control, nutrition, sanitation, and socioeconomic conditions that have enabled more people than ever to live longer and healthier lives. On the other hand, the expansion of older populations introduces new vulnerabilities. It exposes critical gaps in health systems and social protection frameworks that were initially designed for much younger age distributions [10]. For many countries, particularly those in low- and middle-income regions, the demographic shift is occurring at an accelerated pace without the parallel economic growth or institutional strengthening that historically supported aging transitions in wealthier nations [11].

The implications of this transformation extend beyond health into virtually every sector of society. Health systems are being challenged to adapt to a growing burden of chronic, degenerative, and multimorbid conditions that disproportionately affect older adults. Social protection systems are facing mounting pressure as pension schemes, caregiving networks, and intergenerational support structures become increasingly strained by the rising dependency ratios [12,13]. Economically, the shrinking share of the working-age population threatens to slow growth, reduce productivity, and create labor shortages, while simultaneously increasing demand for specialized health and social services. At the societal level, shifts in family structures, urban design, and intergenerational relations are reshaping how communities function, underscoring the urgent need for policies that promote not only longevity but also healthy, functional, and dignified aging [14,15].

Aging is closely associated with a profound shift in the epidemiological profile of populations. Whereas the successful control of infectious diseases defined the 20th century, the 21st century is increasingly characterized by the rising burden of chronic, degenerative, and non-communicable diseases [16]. Cardiovascular conditions, diabetes, chronic respiratory diseases, and neurodegenerative disorders now represent leading causes of morbidity and mortality among older adults. Yet infectious diseases have not disappeared from the landscape of risk [17,18]. In fact, older individuals remain disproportionately vulnerable to respiratory infections such as influenza, pneumococcal disease, and COVID-19, as well as reactivation of latent infections like herpes zoster and tuberculosis. The dual burden of chronic and infectious diseases reflects the complexity of health in aging societies and requires integrated, lifelong approaches to prevention, treatment, and care [19,20,21]. Although Latin American and Caribbean countries are often considered to be in an advanced stage of epidemiological transition, marked by increased life expectancy, declining infectious mortality, and the predominance of chronic noncommunicable diseases, significant regional divergences and country-specific particularities persist. In fact, the region follows a pattern distinct from that of developed countries, characterized by the resurgence of previously controlled infectious diseases and an incomplete transition process. As a result, many countries experience mixed morbidity. This epidemiological polarization, evident not only between countries but also across geographic areas and social classes within the same country, is known as the “prolonged polarized model” [22]. Despite the growing body of evidence, vaccination policies remain primarily focused on childhood, with limited integration into healthy aging strategies.

**Figure 2:**
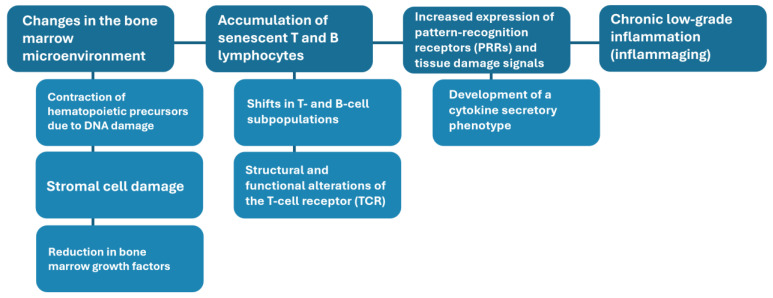
Remodeling of the immune response with aging: from the bone marrow microenvironment to the periphery. TCR, T-cell receptor. PRR: Pattern recognition receptor.

At the core of this vulnerability lies the biological phenomenon of immunosenescence, the gradual deterioration of the immune system with age (Table 1). Immunosenescence is accompanied by inflammaging (Figure 2)—a chronic, low-grade pro-inflammatory state that increases susceptibility not only to infections but also to many age-related chronic diseases (Table 1). Together, these changes weaken both innate and adaptive immune responses, reduce vaccine effectiveness, and contribute to more severe outcomes when infections occur [23,24]. This is compounded by multimorbidity, frailty, and polypharmacy, which further compromise resilience and complicate the management of health conditions in older adults. The result is an escalating demand on healthcare systems: expenditures for individuals aged 65 and older are estimated to be three to five times higher than those for younger adults, and for those aged 80 and older, the gap widens to as much as seven times. These rising costs reflect more extended hospital stays, more frequent use of specialized care, and the need for long-term care services [25].

**Table 1:** Immunological features of inflammaging, underlying biological mechanisms, and associated clinical outcomes [26,27,28].

Immunological Feature	Underlying Biological Mechanisms	Associated Clinical Outcomes
Increased serum TNF-alpha, IL-1β, IL-8, and IL-6	Altered signaling and functional polarization of monocytes and macrophages	Cognitive dysfunction and deterioration of cardiovascular health
Chronic antigenic stimulation derived from pathogens	Chronic antigenic stimulation derived from pathogens. Induction of inflammation driven by pathogen-associated antigenic diversity.	Chronic inflammation and accumulation of visceral fat
Dysbiosis and increased intestinal permeability	Dysbiosis and increased intestinal permeability. Loss of microbiota resilience and impairment of intestinal barrier function.	Chronic inflammation and accumulation of visceral fat
Chronic activation of the inflammasome	Chronic activation of the inflammasome. Proteostasis and loss of inflammasome regulation.	Chronic inflammation and accumulation of visceral fat
Microbial translocation and an increase in tissue-damage molecules	Microbial translocation and increased levels of tissue-damage molecules. Increased exposure to pathogen-associated molecular patterns.	Cognitive dysfunction and deterioration of cardiovascular health
Alteration in liver function, synthesis of inflammatory proteins, and toxicity to the brain, kidney, and muscle	Alteration in liver function, synthesis of inflammatory proteins, and toxicity to the brain, kidney, and muscle. Loss of the capacity to regulate innate inflammation.	Cognitive dysfunction and deterioration of cardiovascular health
Senescent cytokine secretion pattern from the adaptive immune system	Modification of T- and B-cell subpopulations with structural and functional alterations of the TCR	Loss of vaccine responsiveness, vulnerability to infection, and increased risk of cancer and autoimmunity

TCR, T-cell receptor.

In this context, vaccination emerges as a critical, though still underutilized, intervention for promoting healthy aging and extending longevity [29]. Historically, immunization has been mainly perceived as a pediatric intervention, central to protecting children from once-devastating infectious diseases. However, growing scientific evidence now demonstrates that vaccines have essential roles across the entire lifespan, from infancy through advanced age [30,31]. In older adults, vaccines provide direct protection against infections that disproportionately burden this age group and are associated with reduced complications, hospitalizations, and mortality. Emerging evidence also suggests broader immunological and systemic benefits beyond pathogen-specific protection. Influenza and pneumococcal vaccines have been linked to fewer cardiovascular events, while herpes zoster vaccination reduces neuralgia and related declines in quality of life. Overall, vaccination not only prevents acute disease but also helps preserve functional ability and independence in later life [19,32,33,34].

Reframing vaccination as a lifelong health strategy also requires a conceptual shift in public health policies and societal perceptions. In many countries, immunization schedules are still designed primarily for childhood, with limited provision for adolescent, adult, and older adult vaccination. As populations age, this narrow approach fails to capture the potential of vaccines as tools for health promotion, disease prevention, and health system sustainability across the lifespan. A broader perspective recognizes vaccines as one of the few interventions capable of simultaneously preventing disease, reducing healthcare costs, and preserving quality of life for aging populations. This not only enhances individual well-being but also contributes to the sustainability of healthcare systems and the resilience of societies facing demographic change [35].

The WHO’s *Decade of Healthy Ageing (2021–2030)* provides a timely framework to address these intersecting challenges and opportunities (https://www.who.int/initiatives/decade-of-healthy-ageing) (accessed on 1 December 2025). It emphasizes optimizing functional ability, reducing health inequities, and creating environments that support well-being in later life. Within this global agenda, vaccines play a pivotal role, complementing efforts to prevent chronic disease, adapt health systems, and promote social participation. Vaccination is increasingly recognized not only as a biomedical intervention but also as a public health strategy with far-reaching economic and social implications. By preventing disease, reducing dependency, and preserving autonomy, vaccines can help societies navigate the demographic transition with greater equity and sustainability [36,37].

This two-part review, developed by an expert panel, offers a new perspective on the role of vaccines throughout the human lifespan. Part 1 explores the biological foundations of aging and immunity, with emphasis on the mechanisms of immunosenescence and the emerging evidence on vaccine-mediated immunomodulation, and offers partial conclusions grounded in these themes. Part 2 builds on this foundation to examine specific vaccines that contribute to healthier and longer lives, propose vaccination schedules adapted to aging societies, and provide global conclusions that integrate evidence with public health priorities. Together, these reviews aim to reposition vaccination as a cornerstone of lifelong health promotion, a key enabler of functional aging, and a critical pillar of sustainable public health in the 21st century. This work was developed under the coordination of Latin American Society for Vaccinology (SLV), which convened an expert panel from multiple countries in the region to prepare, discuss, and present the findings at an ad hoc meeting in Bogota, Colombia, on 26–27 June 2025 and later at the Latin American Symposium on Maternal Immunization of the SLV, Santo Domingo, Dominican Republic, on 9–10 December 2025, in addition to virtual meetings and coordination efforts. We searched studies and articles in multiple databases, including Web of Science, Scopus, PubMed, SciELO, and ScienceDirect. We were supported by an evidence assessment using the OpenEvidence and VeraHealth platforms; however, this is not a systematic or scoping review.

## 2. Aging and the Immune System

The progressive aging of the global population has profound implications for immune competence, disease risk, and the organization of public health strategies. By 2050, one in six people worldwide will be 65 years of age or older, a demographic shift that compels societies and health systems to reallocate priorities. The challenge is no longer to treat age-related conditions when they appear, but to anticipate them, prevent them, and promote resilience throughout the entire lifespan [38,39]. Central to this effort is the recognition that the immune system, like all biological systems, changes progressively with age. These changes, collectively referred to as immunosenescence, weaken the body’s defense against infections, reduce vaccine effectiveness, and increase the burden of chronic, degenerative, and inflammatory conditions. For this reason, understanding how the immune system ages has become fundamental to predicting clinical outcomes, designing effective therapies, and developing vaccination strategies tailored to older adults [40,41].

### 2.1. Biological Foundations of Immunosenescence

Immunosenescence is a gradual, multifactorial process driven by intrinsic and extrinsic factors. On the intrinsic side, cellular and molecular changes include telomere shortening, genomic instability, mitochondrial dysfunction, and progressive loss of proteostasis. On the extrinsic side, the immune system is affected by the accumulated effects of chronic infections, long-term nutritional patterns, psychosocial stress, and environmental exposures. Together, these factors reshape the architecture of the immune system, reducing its capacity to regenerate, to respond flexibly to novel threats, and to maintain a balance between tolerance and activation [42,43].

Telomere attrition provides a clear example. Each time a cell divides, the protective ends of chromosomes shorten; when they reach a critical point, the cell either stops dividing or enters a senescent state. In lymphocytes, which must proliferate rapidly during immune responses, this shortening has serious consequences. It limits the expansion of naïve T cells, narrows the diversity of the antigen receptor repertoire, and fosters the accumulation of dysfunctional immune clones that contribute to low-grade chronic inflammation [44]. Mitochondrial dysfunction further exacerbates this situation, since energy metabolism becomes less efficient, reactive oxygen species increase, and immune cells lose their ability to mount robust responses. Meanwhile, proteostatic impairments lead to the accumulation of misfolded proteins and toxic aggregates, compromising immune signaling and accelerating inflammatory responses [45].

Epigenetic changes, such as DNA methylation and histone modifications, reprogram gene expression in immune cells. These shifts diminish the plasticity of T and B lymphocytes, restricting their ability to adapt to new antigens and biasing their differentiation toward terminal or exhausted phenotypes. Moreover, environmental stressors, persistent viral infections such as cytomegalovirus, and pollutant exposure can accelerate these epigenetic alterations. The result is an immune system that is simultaneously less effective at fighting new infections and more prone to dysregulated, chronic activation [46].

### 2.2. Remodeling of Innate and Adaptive Immunity

Both innate and adaptive immunity undergo significant remodeling with age, although in distinct but interconnected ways. Within the bone marrow, hematopoietic stem cells accumulate DNA damage and epigenetic changes that impair their self-renewal and differentiation potential. At the same time, stromal cells that provide crucial signals for hematopoiesis produce fewer cytokines, such as interleukin-7 and interleukin-15, resulting in reduced output of lymphoid lineages. The net effect is a microenvironment that is less supportive of immune regeneration and more permissive to the emergence of chronic, low-grade inflammation [47].

Innate immune cells are also affected. Neutrophils show diminished chemotaxis and microbial killing. Macrophages lose phagocytic efficiency and exhibit altered polarization, reducing their ability to coordinate tissue repair. Dendritic cells become less capable of processing and presenting antigens, impairing the activation of adaptive responses. Natural killer cells, although often increased in number, lose their cytotoxic capacity, thereby weakening the body’s first line of defense against viral infections and tumor cells. This paradoxical pattern, characterized by heightened basal inflammatory activity but weaker targeted responses, illustrates one of the most challenging aspects of immune aging [23,42,48].

The adaptive immune system experiences even more profound alterations. Thymic involution, which begins in early adulthood and accelerates with age, drastically reduces the generation of naïve T cells. As a consequence, the T-cell repertoire becomes increasingly dominated by memory and senescent cells. Among CD8+ T cells, highly differentiated subsets accumulate, producing inflammatory mediators but showing poor responsiveness to new antigens. T-cell receptor signaling becomes less effective, and proliferative capacity declines. B cells are also reshaped by aging, with reduced clonal diversity and a greater tendency toward regulatory phenotypes that dampen immune activation but limit the production of high-affinity antibodies. Altogether, these shifts diminish the immune system’s ability to remember past pathogens and to defend effectively against new threats [49].

### 2.3. Inflammaging and Its Clinical Consequences

One of the hallmarks of aging is inflammaging, a chronic state of sterile, low-grade systemic inflammation. This phenomenon emerges from multiple sources. Senescent cells release pro-inflammatory mediators, damaged tissues emit danger-associated molecular patterns, and microbial products may translocate across weakened mucosal barriers. The result is a persistent elevation of cytokines, including interleukin-6, tumor necrosis factor-alpha, and C-reactive protein. Although these mediators are vital in acute defense, their chronic presence gradually damages tissues, impairs repair mechanisms, and fuels degenerative processes [50,51].

Clinically, inflammaging is linked to nearly all primary age-related conditions. It accelerates atherosclerosis, increases the risk of myocardial infarction and stroke, and contributes to the development of insulin resistance and type 2 diabetes. In the nervous system, it promotes neuroinflammation, a critical driver of Alzheimer’s disease and other dementias. Cancer progression is also facilitated by chronic inflammation, which creates a permissive environment for tumor growth and immune evasion. Beyond these conditions, inflammaging undermines resilience to acute infections. During influenza epidemics or the COVID-19 pandemic, older adults experienced disproportionately severe outcomes, reflecting the combined effects of diminished adaptive responses and excessive inflammatory reactions [52]. Frailty, characterized by loss of strength, reduced physiological reserve, and higher vulnerability, has also been directly associated with chronic inflammatory signaling [53,54,55].

### 2.4. Heterogeneity of Immune Aging

Despite the universality of immunosenescence, the pace and consequences of immune aging vary widely among individuals. Genetics, lifestyle, nutrition, infections, and socioeconomic conditions all shape its trajectory. Some individuals exhibit “slow immune aging,” maintaining diverse antigen repertoires and effective responses well into advanced age. Others experience “accelerated immune aging,” in which immune decline appears earlier and more severely, predisposing them to multimorbidity, disability, and early mortality [56].

The concept of “immune age” has therefore gained prominence. Unlike chronological age, immune age seeks to capture the biological state of the immune system by measuring biomarkers such as telomere length, epigenetic signatures, transcriptomic profiles, and immune cell phenotypes. This perspective enables a more nuanced evaluation of vulnerability and resilience and has the potential to inform individualized vaccination and preventive strategies. Recognizing that immune aging is dynamic and partly modifiable also underscores the role of interventions such as physical activity, balanced nutrition, control of chronic infections, and stress reduction in delaying immune decline [57].

### 2.5. Public Health Implications

The remodeling of the immune system with age has significant implications for public health. Vaccines that are highly effective in children and young adults often elicit weaker responses in older adults, resulting in reduced protection. This challenge has spurred the development of new strategies, including high-dose formulations, vaccines with novel adjuvants that enhance antigen presentation, and antigen conjugation technologies that improve immunogenicity (Table 2). The concept of “trained immunity,” which involves reprogramming innate responses through epigenetic changes, is an emerging field that could also enhance vaccine efficacy in aging populations [58].

Preserving immune fitness across the lifespan requires a broad approach. Vaccination remains essential, but it should be complemented by lifestyle modifications, management of chronic diseases, and interventions that reduce systemic inflammation. By mitigating both immunosenescence and inflammaging, it is possible not only to reduce infectious disease burden but also to influence the onset and progression of non-communicable diseases. In this sense, the immune system becomes a central target of health promotion, a mediator of healthy longevity, and a key determinant of whether aging populations will remain active, independent, and socially engaged [59].

### 2.6. Vaccine-Mediated Immunomodulation

The progressive aging of populations worldwide has placed vaccination at the center of discussions on how to promote health and resilience across the lifespan. In younger individuals, vaccines are primarily valued for their role in preventing specific infections. In older adults, however, vaccines assume a dual role: they continue to prevent acute infectious diseases but also modulate immune function, mitigating the effects of immunosenescence and chronic inflammation. This broader perspective recognizes that vaccines can do more than protect against pathogens; they can also help maintain immune fitness, reduce frailty, and preserve quality of life [23,58].

**Table 2:** Strategies to enhance vaccine-induced immune responses in older adults: benefits and areas for improvement [60,61,62].

Strategy or Platform	Vaccine, Technique, or Component	Expected Benefit	Clinical Impact	Limitations/Areas for Improvement
mRNA platforms	COVID-19 vaccines	Induction of robust T-cell and B-cell immune responses.	Prevention of severe disease and reduction in infection-related hyperinflammation.	The immune response wanes relatively rapidly, necessitating booster doses.
High-dose antigen formulations	Influenza vaccines, live-attenuated varicella-zoster vaccine.	Increased antigen visibility, leading to higher antibody titers.	Improved antibody titers and enhanced pathogen-specific cellular immunity; superior prevention of hospitalization and mortality compared with standard-dose vaccines.	Higher cost and limited antigen availability; waning efficacy over time; dose-dependent association with cardiovascular events reported for influenza vaccines.
Protein conjugation of pneumococcal capsular polysaccharides	PCV13, PCV15, PCV20, PCV21	Enhanced induction of memory B cells and higher antibody concentrations compared with non-conjugated polysaccharide vaccines.	Reduction in hospitalizations and mortality from invasive pneumococcal disease and community-acquired pneumonia.	Not all conjugate vaccines are available for older adults in national immunization programs across all countries.
Use of novel adjuvants	MF59, AS01B	Increased local cytokine production at the injection site, improving recruitment and activation of innate immune cells and antigen presentation.	Enhanced humoral and antigen-specific cellular immune responses.	May be associated with increased local reactogenicity.

PCV, Pneumococcal conjugate vaccine; mRNA, messenger ribonucleic acid; MF59, Squalene-based oil-in-water vaccine adjuvant; AS01B, Liposome-based adjuvant system containing MPL and QS-21.

While increasing evidence suggests that certain vaccines may influence immune homeostasis and inflammatory pathways, it is important to emphasize that the concept of vaccine-mediated immunomodulation in aging remains heterogeneous and incompletely defined [63]. At present, only selected mechanisms and clinical effects are supported by consistent empirical data, whereas many proposed pathways, particularly those related to immune rejuvenation, long-term modulation of inflammaging, or systemic anti-aging effects, remain largely exploratory [64,65]. Consequently, vaccines should primarily be regarded as established tools for infection prevention, with potential immunomodulatory effects that are context-dependent and still under active investigation [41,66].

### 2.7. From Simple Protection to Systemic Effects

Historically, vaccination was seen as a targeted intervention that generated immunity against a specific pathogen, thereby reducing incidence, morbidity, and mortality. While this remains true, increasing evidence suggests that vaccines have broader effects on the immune system and overall health. In older adults, immunization against influenza, pneumococcus, or herpes zoster not only prevents infection but also reduces hospitalizations and mortality from complications such as pneumonia, myocardial infarction, and stroke. These benefits extend beyond pathogen-specific protection, suggesting that vaccines may influence the risk or severity of certain age-related conditions, mainly through an indirect mechanism [32,67].

For example, influenza vaccination has been associated with a lower risk of cardiovascular events, whereas pneumococcal vaccines reduce the incidence of invasive infections that can accelerate frailty and disability. Herpes zoster vaccination not only prevents shingles but also reduces the chronic pain and depression associated with post-herpetic neuralgia. By preventing these events, vaccines help preserve independence and delay the onset of functional decline. The clinical implications are clear: vaccination reduces not only the burden of infection but also the cascade of consequences that amplify morbidity in aging populations [68,69,70,71,72].

However, although these associations are biologically plausible and supported by growing observational evidence, they should not be interpreted as definitive proof of direct systemic or anti-aging effects. Many of the reported benefits are likely mediated indirectly by preventing acute infections and their downstream complications, rather than directly through modification of aging pathways. Further randomized, mechanistic studies are required to clarify the extent to which these outcomes reflect causal immunomodulatory effects [73,74].

### 2.8. Vaccines and the Inflammatory Burden

Aging is characterized by a background of chronic low-grade inflammation, often referred to as inflammaging. This state not only drives many degenerative diseases but also interferes with effective immune responses. Vaccination may help modulate the inflammatory burden, primarily by preventing infection-related inflammatory episodes. By preventing infections, vaccines reduce episodes of acute inflammation that would otherwise exacerbate inflammaging. Moreover, some vaccines appear to exert regulatory effects on immune pathways, helping restore balance between pro-inflammatory and anti-inflammatory responses [75].

The reduction in systemic inflammation has long-term benefits. Chronic inflammatory stress contributes to atherosclerosis, type 2 diabetes, neurodegeneration, and cancer. By reducing the number of infectious triggers and, indirectly, inflammatory signaling, vaccines contribute to healthier aging trajectories. In this sense, they can be understood as interventions that influence both the infectious and non-infectious dimensions of disease [76].

### 2.9. Immunobiography and Heterogeneity of Responses

Not all older adults respond to vaccines in the same way. The variability of immune responses reflects an individual’s “immunobiography,” a concept that describes the cumulative history of infections, vaccinations, environmental exposures, and lifestyle factors that shape the immune system throughout life. Some individuals enter old age with relatively preserved immune responsiveness, while others display accelerated immune decline. Vaccination outcomes mirror these differences, with some older adults mounting strong protective responses and others showing limited benefit [77].

This variability has important implications for public health and clinical practice. It emphasizes that vaccine strategies for older adults must be designed with flexibility, utilizing tools that enhance immunogenicity in individuals with diminished immune responses. It also highlights the need for life-course vaccination policies, since immune trajectories are influenced by exposures long before old age. Sustained and timely immunization across childhood, adulthood, and middle age can help preserve immune fitness and reduce the severity of immunosenescence later in life [78].

### 2.10. Trained Immunity and Epigenetic Reprogramming

One of the most promising areas of research on vaccine-mediated immunomodulation concerns the concept of trained immunity. Traditionally, innate immunity was considered non-specific and lacking memory. However, evidence suggests that vaccines can induce long-lasting functional changes in innate immune cells, including monocytes, macrophages, and natural killer cells. These changes are mediated by epigenetic reprogramming and metabolic shifts that increase responsiveness to subsequent challenges [64,79].

The Bacillus Calmette–Guérin (BCG) vaccine is the most studied example (Table 3). Beyond its role in tuberculosis prevention, it enhances resistance to unrelated infections by training innate cells to respond more effectively. While most studies have been conducted in children or young adults, harnessing trained immunity in older populations is highly attractive (Table 3). If vaccines can be designed to induce beneficial epigenetic modifications in innate immune cells, they could help compensate for age-related declines in adaptive responses [80].

**Figure 3:**
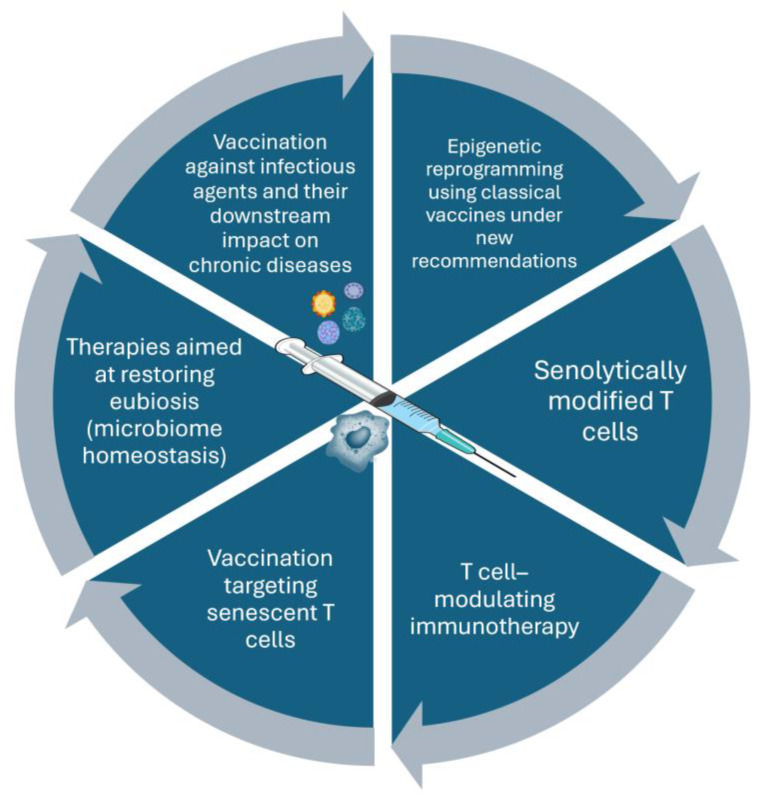
Conceptual model of potential pathways for restoring immune fitness during aging. The figure illustrates theoretical mechanisms through which vaccination, adjuvanted formulations, trained immunity, lifestyle interventions, and emerging immunotherapeutic approaches may contribute to the partial restoration of immune function in older adults. These pathways include modulation of innate and adaptive immune responses, reduction in chronic inflammation, and enhancement of immune resilience. The model is intended to be hypothesis-generating and reflects current experimental and translational research rather than established clinical outcomes. **Immunotherapy**: Use of drugs that modulate T-cell activation and proliferation, including calcineurin inhibitors, monoclonal antibodies, and immune checkpoint inhibitors. **Senolytically modified cells**: Use of CAR-T cell-based therapies targeting senescent cells. **Vaccination against senescent T cells**: Vaccine strategies targeting senescence-associated antigens, such as CD153.

This research area offers opportunities for a new generation of vaccines that not only elicit antibody and T-cell responses but also rejuvenate innate immunity (Figure 3). Such vaccines would act as broad modulators of immune fitness, offering heterologous protection against a range of pathogens and potentially reducing chronic inflammation (Table 3) [81].

### 2.11. Innovative Approaches to Enhance Vaccine Responses

Given the limitations imposed by immunosenescence, a variety of innovative strategies have been developed to improve vaccine efficacy in older adults [82,83]. These include:

- **Adjuvants**: Modern adjuvants such as MF59 and AS01 enhance antigen presentation and promote stronger T- and B-cell responses. Their inclusion in vaccines for older adults has demonstrated improved immunogenicity.
- **Higher antigen doses**: High-dose influenza vaccines provide greater antigen exposure, compensating for the reduced responsiveness of aging immune systems and improving clinical protection.
- **Conjugate vaccines**: By linking polysaccharides to protein carriers, conjugate vaccines recruit T-cell help and generate stronger, longer-lasting antibody responses.
- **Pattern recognition receptor agonists**: Incorporating molecular motifs that mimic microbial signals enhances the activation of dendritic cells and other antigen-presenting cells.
- **Epigenetic modulation**: Experimental approaches aim to deliberately induce beneficial epigenetic changes, effectively “resetting” immune cells to a more functional state.

These innovations demonstrate a paradigm shift: vaccines are no longer viewed merely as prophylactic agents against infection but as sophisticated tools capable of remodeling immune function. While not all strategies have yet translated into large-scale clinical benefits, the trajectory of research is clear. Vaccinology is shifting toward precision approaches that accommodate the biological realities of aging [84,85].

**Table 3:** Promising mechanisms for modulating vaccine-induced immune responses [62,86,87,88].

Mechanism	Potential Application	Proposed Mechanism of Action	Expected Outcome	Unresolved Issues/Gaps
Vaccine-induced epigenetic modifications via trained immunity	Novel immunomodulatory indications for BCG and measles vaccines, including enhancement of responses to unrelated pathogens and vaccines	Induction of trained immunity through pattern recognition receptor signaling pathways (e.g., NOD2), histone H3 modification, and metabolic reprogramming of hematopoietic stem cells toward progeny with a protective immunophenotype	Modulation of metabolic pathways with increased production of trained myeloid cells and monocytes; positive heterologous effects on overall mortality, cognitive development, and cancer incidence	Additional clinical studies are required to support recommendations beyond pediatric populations
Implementation of novel correlates of protection	Improved assessment of immunogenicity of inactivated influenza vaccines in older adults	Measurement of IFN-γ/IL-10 ratios, granzyme B levels, and functional antibody-dependent cellular cytotoxicity (ADCC) assays	Improved correlation between immune markers and vaccine effectiveness in older adults	Challenges in biomarker standardization and implementation across laboratories
Incorporation of pathogen-associated molecular patterns (PAMPs) as PRR ligands in vaccine formulations	Use of advanced adjuvants to enhance cellular immune responses	Use of adjuvants such as monophosphoryl lipid A and synthetic glucopyranosyl lipid derivatives to promote cross-presentation and cytotoxic T-cell activation	Induction of strong cellular effector immune responses	Further research is required before routine clinical use

PRR, Pattern recognition receptor. PAMP, Pathogen-associated molecular pattern. NOD2, Nucleotide-binding oligomerization domain-containing protein 2. IFN-γ, Interferon gamma. IL-10, Interleukin-10. ADCC, Antibody-dependent cellular cytotoxicity. BCG, Bacillus Calmette–Guérin vaccine.

### 2.12. Lifelong Immunomodulation

Vaccination should be understood as a lifelong practice, much like exercise or nutrition, that contributes to healthy aging. Immunization is not limited to childhood; it continues to confer benefits throughout adulthood and into old age. By reinforcing immune fitness, vaccines reduce infections and may indirectly influence the course of chronic diseases and functional decline. Significantly, they reduce healthcare costs by preventing hospitalizations, long-term complications, and premature dependency [84,89].

Integrating vaccination into the broader concept of lifestyle medicine requires public health systems to adapt their strategies. Education, accessibility, and policy support are crucial to ensuring that vaccines are consistently administered throughout a person’s life. In aging societies, this approach is critical not only for individual well-being but also for social and economic sustainability. By preserving independence in older adults, vaccination reduces the burden on caregivers, healthcare infrastructure, and social security systems [90].

### 2.13. Future Directions

Emerging lines of research are pushing the boundaries of what vaccine-mediated immunomodulation can achieve. Some studies are exploring vaccines targeting senescent immune cells or antigens associated with cellular aging to rejuvenate the immune system [91]. Others are examining the interactions between vaccines and the gut microbiota, recognizing that microbial communities strongly influence systemic immunity. There is also increasing interest in the possibility of vaccines designed to potentially influence pathways involved in chronic inflammatory diseases, such as Alzheimer’s disease [53]. Recent studies indicate that vaccines against respiratory syncytial virus (RSV) and herpes zoster may confer protective effects beyond infection prevention, being associated with a reduced risk of cognitive impairment and neurodegenerative diseases, possibly by mitigating systemic inflammation, viral reactivation, and immune-mediated neural damage [92].

These developments, although still experimental, illustrate a profound conceptual evolution. Vaccines are shifting from agents of pathogen-specific defense to instruments of immune health management across the lifespan. If successful, such innovations would redefine vaccination as a central pillar of healthy longevity, with benefits extending far beyond infection control [25,42,47].

### 2.14. Conceptual Implications

Ultimately, vaccine-mediated immunomodulation reframes how vaccination is perceived in medicine and public health. Vaccines not only prevent acute infections, but they also shape the long-term trajectory of immune aging. They influence how individuals experience frailty, chronic disease, and independence. In this sense, each vaccination contributes to an individual’s immunobiography, leaving lasting marks on immune resilience [50].

From this broader perspective, vaccination can be seen as a determinant of healthy longevity. It is never too late to vaccinate, but it is far more effective to begin early and sustain immunization throughout life [93]. By doing so, societies can anticipate the challenges of demographic change, reduce health inequities, and promote aging that is not only longer but also healthier, more independent, and more dignified [1,13,30,38].

## 3. Related Public Health Strategies

Translating lifelong vaccination concepts into effective public health action requires context-sensitive and sustainable implementation models. Several health systems have begun to integrate adult and older adult immunization into primary care, chronic disease management, and preventive health programs. For example, some high-income countries have incorporated influenza, pneumococcal, herpes zoster, and COVID-19 vaccines into routine geriatric assessments, electronic health record reminders, and pharmacist-led vaccination services, thereby improving coverage and continuity of care [94,95].

In middle-income settings, opportunistic vaccination strategies linked to outpatient visits, non-communicable disease clinics, and social insurance programs have demonstrated feasibility and cost-effectiveness by leveraging existing healthcare infrastructure. Community-based outreach, mobile vaccination units, and integration with social protection programs are additional approaches to improving access for underserved populations [96,97].

Economic evaluations suggest that adult vaccination programs can be cost-effective by reducing hospitalizations, long-term disability, and healthcare utilization associated with preventable infections. However, successful implementation requires sustained financing, workforce training, reliable supply chains, and public engagement strategies to address vaccine hesitancy [98,99].

Future policy development should prioritize life-course immunization registries, integrated financing mechanisms, and adaptive delivery models tailored to demographic and health system capacities [100].

## 4. Limitations

This review has several important limitations. First, it is a narrative synthesis rather than a systematic review and therefore does not follow a predefined protocol for study selection, quality appraisal, or evidence grading. As a result, the strength and consistency of the available evidence vary across topics, and the conclusions should be interpreted with appropriate caution.

While the protective effects of vaccines against infections, hospitalizations, and mortality are supported by robust randomized and observational data, much of the evidence for broader benefits—such as reductions in cardiovascular events, frailty, cognitive decline, or functional impairment—derives predominantly from observational studies, secondary analyses, and mechanistic investigations. These designs are inherently susceptible to residual confounding, selection bias, and healthy-user effects; therefore, they primarily support associative rather than causal inferences.

Moreover, this review did not formally apply standardized frameworks for grading the quality or certainty of evidence, such as GRADE or similar approaches. Consequently, differences in methodological quality, sample size, follow-up duration, and risk of bias across studies were not systematically weighted. Future systematic reviews and meta-analyses using formal evidence appraisal methods will be essential to clarify the magnitude and causality of these associations.

Emerging concepts, including trained immunity and vaccine-mediated immunomodulation in older adults, remain under active investigation, and their long-term clinical relevance has yet to be fully established in large, randomized trials. In addition, most available data originate from high-income settings, which may limit generalizability to low- and middle-income countries with different epidemiological profiles, healthcare infrastructures, and vaccination policies.

The heterogeneous nature of aging, comorbidities, and immunobiography among older adults further complicates the interpretation of vaccine-related outcomes. Well-designed prospective studies and randomized controlled trials are needed to better delineate causal pathways and to determine which populations derive the greatest non-specific benefits from vaccination.

Finally, the conceptual framework presented in this review reflects an evolving field, and some interpretations of vaccine-related modulation of immune aging should be viewed as hypothesis-generating rather than definitive.

## 5. Conclusions

The demographic transition of the 21st century is reshaping global health priorities, as population aging exposes vulnerabilities driven by immunosenescence and inflammaging, increasing the burden of infectious and chronic diseases. Aging involves complex immune remodeling that affects innate and adaptive responses, reduces pathogen defense and vaccine effectiveness, and promotes chronic inflammation, contributing to multimorbidity and frailty.

Vaccination is a powerful intervention across the lifespan, extending beyond childhood prevention. In older adults, vaccines not only prevent high-risk infections but may, in selected contexts, influence immune homeostasis and systemic inflammation. Observational evidence linking vaccination to reduced hospitalizations, cardiovascular events, disability, and functional decline supports its role as a cornerstone of healthy longevity.

The concept of vaccine-mediated immunomodulation further broadens this view, recognizing vaccines as tools that can reprogram immune function and counter aspects of immunosenescence through mechanisms such as trained immunity, epigenetic remodeling, and advanced adjuvants. This reflects a shift toward vaccines as dynamic instruments of immune health management.

Despite these advances, challenges persist due to heterogeneous vaccine responses among older adults, shaped by individual immunobiographies. Public health strategies must therefore adopt a lifelong, flexible, and equitable approach to immunization. Overall, vaccines are essential not only for preventing infection but also for promoting functional aging and health system sustainability. Part 2 will address specific vaccines, tailored schedules, and global perspectives to further position vaccination as a pillar of lifelong health.

## Data Availability

Not applicable.
